# Left Lower Lung Collapse in a Patient Undergoing Endoscopic Procedure

**DOI:** 10.1155/2020/8670102

**Published:** 2020-01-31

**Authors:** Akshatha Kamath, Joel Yarmush, Sneha Rao

**Affiliations:** Department of Anesthesiology, NYP-Brooklyn Methodist Hospital, New York City, NY, USA

## Abstract

ASA closed claims from 2000 to 2009 have shown that adverse respiratory events are more common in nonoperating room locations like endoscopy suite than in the operating room (44% v/s 20%). Here, we report a case of lung atelectasis which resulted in hypoxemia in a malnourished patient undergoing endoscopic procedure. It is crucial to identify the high-risk patients and monitor them appropriately in the postoperative phase. Continuous capnometry may offer additional benefit by identifying hypercapnia, hypoventilation at the earliest in the recovery area, thus preventing serious complications.

## 1. Introduction

Patients after Roux-en-Y gastric bypass often present with stomal strictures, and PEG placements are made in an attempt to improve their nutrition. Endoscopic balloon dilatation of the stricture is a common procedure used to treat this condition and has a success rate of 95% [1]. Respiratory fatigue under monitored anesthesia with sedation can lead to profound respiratory morbidity in severely malnourished patients. They can present with central apnea secondary to sedatives and ventilation perfusion mismatch leading to hypoxia. We present a rare case of pulmonary complication in a patient undergoing such procedure.

## 2. Case Report

**Figure 1:**
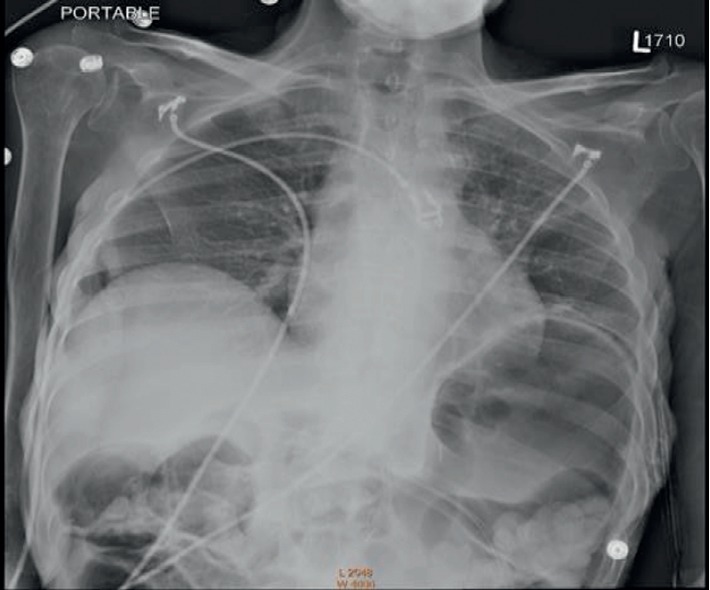
CXR showing the left lower lobe atelectasis and gastric distension.

A 51-year-old female with a past medical history of asthma, peptic ulcer disease, and severe malnutrition was scheduled for elective balloon dilatation of gastro-gastric fistula in our endoscopy unit. The patient had Roux-en-Y bypass for morbid obesity in the past. A percutaneous gastrostomy tube placement was attempted but unsuccessful due to her difficult anatomy with a subsequent laparoscopically placed gastrostomy tube. During her present admission, she complained of persistent symptoms of abdominal pain and poor oral intake and had developed an abscess around the G-tube site requiring a repeat upper endoscopy. Preoperative assessment was significant for severe debilitation with a BMI of 17. Airway exam was within normal limits. Physical exam was significant for muscle loss with poor skin turgor. She was classified as ASA III. Most recent labs were significant for anemia with a hematocrit of 29.4, GFR >90 ml/min, and normal electrolytes. Standard ASA monitors were applied, and nasal cannula was connected to an oxygen flow of 3 L/min. 0.5 ml benzocaine 20% spray to the oropharynx was used for topical anesthesia. Intravenous sedation was maintained with intermittent doses of propofol to a total of 180 mg. The procedure lasted for an hour. Attempts were made to balloon dilate the gastro-gastric fistula at 15 mm without much success. Vitals remained stable through the major part of the procedure. However, towards the end of the procedure, shallow respirations were seen and oxygen saturation dropped to 80 s, suggesting impending respiratory arrest. She was immediately rescued with positive pressure ventilation by using an Ambu-bag mask. After almost ten minutes of positive pressure ventilation, oxygen saturation began to stabilize to low at 90 s and she was beginning to arouse. Respiratory exam showed absent breath sounds in the left lung base with no crackles or wheeze. Abdomen appeared distended. An immediate chest X-ray was obtained, which showed an extremely distended stomach with left lower lobe collapse. This had partially improved with positive pressure ventilation by the time chest X-ray was taken (Figure 1).

**Figure 2:**
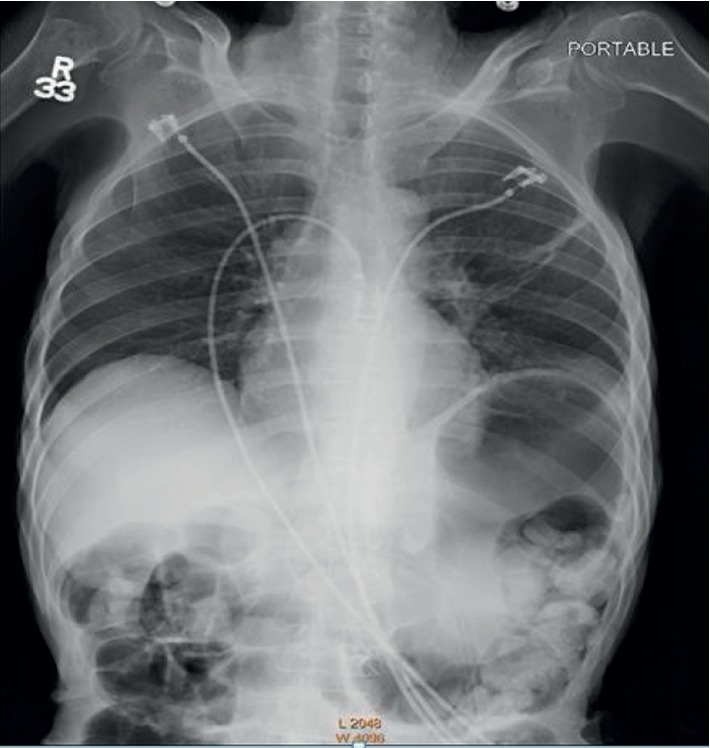
Repeat CXR showing left lung expansion.

Subsequently, she was treated with high-flow oxygen via a non-rebreather bag, incentive spirometry, and simethicone which improved her oxygen saturations and pass flatus over the next 4 hours. A repeat chest X-ray showed some expansion of left lung volume and further improvement in oxygen saturations to 94% (Figure 2). She was admitted under GI service for overnight observation.

## 3. Case Discussion

Cardiopulmonary events (0.9%) constitute major proportion of endoscopy-associated complications [2]. This has been attributed to multiple causes like dose of sedative medications, speed of administering, patient ASA status, and past cardiopulmonary problems [3]. Studies have shown that patients with advanced age and those requiring greater amount of propofol tend to need active interventions to prevent hypoxemia. Other risk factors for desaturation include BMI and procedure time [4]. The American Society of Anesthesiology (ASA) and American Society of Gastrointestinal Endoscopy (ASGE) guidelines suggest that all patients undergoing deep sedation have pulse oximetry, blood pressure, EKG, and capnography monitored [5].

The likely mechanisms for desaturation during endoscopy are hypoventilation and apnea from sedation, decreased diaphragmatic performance leading to reduced functional residual capacity, respiratory fatigue in the malnourished, and ventilation perfusion mismatch. Gastric insufflation which is commonly used in these procedures can cause respiratory compromise secondary to hypoinflation of the left lung leading to V/Q mismatch. Other possibilities to be considered are pneumothorax, pneumomediastinum, methemoglobinemia, lung collapse, or atelectasis from mucus plugs, bronchospasm, gastric aspiration, or partial obstruction of the airway by the endoscope [6–8].

Lung atelectasis is a common finding in patients undergoing general or sedation anesthesia regardless of the route of anesthetic administration (intravenous or inhalational) and method of ventilation (spontaneous or controlled) [9]. There have been numerous studies showing the complications of respiratory depression like desaturation, hypercarbia, respiratory acidosis, hyperkalemia, myocardial depression, and arrhythmias during conscious sedation [7, 8].

In a study by Bell et al. [10], 100 consecutive patients undergoing routine upper endoscopy were sedated with intravenous midazolam (average dose 6.3 mg). Seven percent had oxygen saturation fall below 80% during the procedure, although no serious long-term complications were reported. The use of a combination of a benzodiazepine and an opioid seems to increase respiratory depression during endoscopy modestly. Administration of supplemental oxygen during endoscopy to prevent significant desaturation has been studied and proved to be beneficial. This explains that desaturation is due to V/Q mismatch rather than shunt or hypoventilation [11–13]. Supplemental oxygen, however, does not affect carbon dioxide (CO_2_) levels in the bloodstream, and standard pulse oximetry does not measure hypoventilation or hypercarbia. Freeman and colleagues analyzed 101 endoscopic procedures for a relationship between hypoxemia and hypercapnia. Among their findings was that normalization of pulse oximetry by means of supplemental oxygen masked the degree of hypercapnia [14]. Nelson et al. evaluated adding transcutaneous CO_2_ monitoring during endoscopy and compared it with standard monitoring. Although there were significantly fewer episodes of severe CO_2_ retention in the study group versus the control group, there were no clinically significant differences between the two groups [15].

Lam et al. describe the importance of continuous capnometry and pulse oximetry in the postoperative area, which helps in early detection of any postoperative respiratory depression as the desaturation could be lately appearing in case of supplemental oxygen administration [16].

Endoscopy is increasingly being done for diagnostic and therapeutic purposes. High-risk patients should be identified at the preprocedural visits by the gastroenterologist and anesthesiologist to determine if they will tolerate the procedure. A patient with malnutrition, end-stage lung disease, or cardiac disease may not handle the procedure as well as their healthy counterparts. Careful assessment of potential risks and benefits and alternatives is essential before initiating the procedure. Such patients should be optimized at their best prior to the procedure. Optimization may include chest physiotherapy, bronchodilators, incentive spirometry, and antibiotics for any evidence of ongoing infection. Smoking cessation should be encouraged.

## 4. Conclusion

Intravenous sedation and monitored anesthesia remain the anesthetic of choice for most endoscopic procedures. Sedation can be a challenging task, and patients can go into deeper-than-intended plane of anesthesia. The anesthesiologist has to deal with unpredictable drug responses, patient comorbidities, and history of substance abuse. Specific antidotes for opiates and benzodiazepines should be available to rescue patients from severe respiratory depression. Prompt recognition of adverse events and appropriate management are necessary for optimal outcomes. Emphasis must be provided to match the patient, procedure, and provider settings.
